# CABG and Preoperative use of Beta-Blockers in Patients with Stable Angina are Associated with Better Cardiovascular Survival

**DOI:** 10.21470/1678-9741-2017-0138

**Published:** 2018

**Authors:** Victor Dayan, Diego Perez, Eloisa Silva, Gerardo Soca, Jorge Estigarribia

**Affiliations:** 1 Instituto Nacional de Cirugía Cardíaca, Montevideo, Uruguay.

**Keywords:** Coronary artery bypass, Adrenergic betaantagonists/therapeutic use, Angina, stable, Preoperative Care/methods

## Abstract

**Objective:**

In contrast to unstable angina, optimal therapy in patients with stable angina is debated. Our aim was to evaluate the outcomes of patients with stable angina scheduled for isolated coronary artery bypass grafts and the effect of preoperative use of beta-blockers. Overall and cardiovascular survivals were our primary outcome. Operative mortality and postoperative complications along with subgroup analysis of diabetic patients were our secondary outcomes.

**Methods:**

Retrospective evaluation of patients with stable angina scheduled for isolated coronary artery bypass grafts was included. Pre- and postoperative variables were extracted from the institution database. Survival was obtained from the National Registry.

**Results:**

We included 282 patients with stable angina, with a mean age of 65.6±9.5 years. 26.6% were female and 38.7% had diabetes. Three-vessel disease was present in 76.6% of patients. Previous beta-blocker treatment was evident in 69.9% of patients. 10-year overall survival in the whole population was 60.5% (95% confidence interval [CI]: 50.3-70.7%). Operative mortality during the study period was 3.5%. Patients with preoperative use of beta-blocker therapy had better overall survival (9.0 years, 95%CI: 8.6-9.5) than those without treatment (7.9 years, 95%CI: 7.1-8.8 years; *P*=0.048). Predictors for overall survival were: hypertension, diabetes, and age. Predictors for cardiovascular survival in diabetic patients were: beta-blocker use, gender, and age.

**Conclusion:**

Coronary artery bypass grafts surgery in patients with stable angina carries low operative mortality, postoperative complications, and excellent long-term cardiovascular survival. The preoperative use of beta-blockers in diabetic patients is associated with better cardiovascular survival after coronary artery bypass grafts.

**Table:** 

Abbreviations, acronyms & symbols	
ACS	= Acute coronary syndromes
AMI	= Acute myocardial infarction
CABG	= Coronary artery bypass grafts
CAD	= Coronary artery disease
CI	= Confidence interval
COPD	= Chronic obstructive pulmonary disease
CPB	= Cardiopulmonary bypass
HR	= Hazard ratio
IMA	= Internal mammary artery
INCC	= Instituto Nacional de Cirugía Cardíaca
OPCABG	= Off-pump coronary artery bypass graft
PCI	= Percutaneous coronary intervention
PTCA	= Percutaneous transluminal coronary angioplasty
SD	= Standard deviation
TTFM	= Transit time flow measurement

## INTRODUCTION

Patients with stable angina (SA) represent the largest group of patients with coronary artery disease (CAD). These patients may be treated with medical therapy alone or in combination with revascularization by either coronary artery bypass grafts (CABG) or percutaneous coronary intervention (PCI)^[1]^. Multiple randomized controlled trials have compared the efficacy of revascularization *versus* optimal contemporary medical therapy^[2-4]^. Although these studies have shown no difference between them, their main drawback is the restrictive recruitment strategies and unrealistic levels of medication compliance and lifestyle modification^[5]^. Thus, there is substantial uncertainty as to the generalizability of randomized control trials findings to routine clinical practice. On the other hand, recent metaanalysis have highlighted improved survival in patients after revascularization^[6,7]^.

The main arguments against CABG in patients with stable angina are operative mortality and postoperative complications. We aimed to study 5 and 10-year survivals, postoperative complications and predictors of survival in a local cohort of patients with stable angina scheduled for isolated CABG (primary outcome). Furthermore, we evaluated the effect of preoperative use of beta-blockers and performed subgroup analysis with diabetic patients (secondary outcome).

## METHODS

### Patients

The study was approved by the Instituto Nacional de Cirugía Cardíaca (INCC) review board and informed consent was waived due to its retrospective nature. Patients with stable angina who underwent isolated CABG from January 2006 and December 2014 were included. Exclusion criteria used were: emergency/urgency and left main stenosis. During that period, 352 patients underwent surgery for isolated stable angina. From these, 70 patients had left main stenosis or were operated under emergency/urgency basis. We included 282 patients with stable angina who underwent isolated CABG in a non-urgent or emergent basis.

Basal demographic characteristics, postoperative outcomes (operative mortality, hemodialysis, pneumonia, and stroke), and survival were included for each patient.

Operative mortality was defined as death 30 days after surgery or during primary hospitalization. Postoperative complications are defined as those which arise during the first 30 days after surgery. At discharge, all patients were prescribed with the same medication protocol which is adjusted individually. Basically, all patients receive statins, beta-blockers, and aspirin. Adherence to medication could only be evaluated after one-month of surgery (when the last clinical control was performed) and 100% of patients adhered to the prescription.

Follow-up was achieved in 100% of patients. Mortality data was obtained through the governmental agency National Resources Fund (Fondo Nacional de Recursos).

### Surgery

A median sternotomy was performed in all patients. CABG surgery was performed with cardiopulmonary bypass (CPB) in a standardized fashion using ascending aortic cannulation and two-stage venous cannulation of the right atrium. Intermittent cold crystalloid Buckberg cardioplegia was delivered antegrade via the aortic root. Off-pump or beating heart procedures were performed achieving stabilization using Medtronic Octopus and Starfish stabilizers. Grafting was attempted on all vessels measuring 1 mm or more in diameter with a 50% or greater stenosis.

Graft patency was measured using transit time flow measurement (TTFM) (VeriQ, Medisitim) as previously described by D'Ancona et al.^[8]^. Measurements were performed after weaning of CPB and with systolic blood pressure between 120-140 mmHg. Revision of anastomosis was performed when pulsatility index was higher than 5.

### Outcomes

Overall and cardiovascular survivals were our primary outcome. Operative mortality and postoperative complications along with subgroup analysis of diabetic patients were our secondary outcome. Impact of multiple bypass using internal mammary artery (IMA) was evaluated comparing survival in patients who received one or more than one IMA bypass.

### Statistical Analysis

Continuous variables were expressed as mean ± standard deviation (SD) and compared using Student t-test. Categorical variables were expressed as absolute numbers (%) and compared using Chi-square test. Survival was analyzed with Kaplan-Meier and log-rank test. Cox regression was used to evaluate independent predictors for overall and cardiovascular survivals. Variables with *P*<0.1 after univariate analysis were entered in the multivariate analysis.

## RESULTS

Mean age was 65.6±9.5 years; 26.6% were female and 38.7% had diabetes. Previous percutaneous transluminal coronary angioplasty (PTCA) was present in 14.9% of patients and 76.6% of patients had three-vessel disease. Previous beta-blocker treatment was evident in 69.9% of patients (Table 1). Pre- and operative variables were similar between patients with and without beta-blockers, except for higher incidence of chronic obstructive pulmonary disease (COPD) and off-pump coronary artery bypass graft (OPCABG) in the latter group (Table 2).

**Table 1:** Basal demographics of included patients (n=282).

Variable	Patients (282)
Age (SD)	65.6 (9.5)
Female (%)	75 (26.6)
Smoker (%)	71 (25.2)
Hypertension (%)	222 (78.7)
Diabetes (%)	109 (38.7)
Dyslipidemia (%)	243 (86.2)
PVD (%)	19 (6.7)
COPD (%)	7 (2.5)
Stroke (%)	11 (3.9)
AMI (%)	__
Preoperative creatinine (mg/dl)	1.4 (5.5)
LVEF (%)	55.3 (12.1)
Previous PTCA (%)	42 (14.9)
Previous CABG (%)	3 (1.1)
NYHA III-IV (%)	18 (11.4)
Angina CCS III	67 (25.3)
Vessel disease (%)	
1	23 (8.1)
2	43 (15.2)
3	216 (76.6)
>1 IMA bypass (%)	75 (26.5)
OPCABG (%)	57 (20.2)
Beta-blockers (%)	197 (69.9)
Statins (%)	84 (29.8)
Anti-aggregation (%)	233 (82.6)

AMI=acute myocardial infarction; CABG=coronary artery bypass grafts; CCF=Canadian Cardiovascular Society; COPD=chronic obstructive pulmonary disease; IMA=internal mammary artery; LVEF=left ventricular ejection fraction; NYHA=New York Heart Association; OPCABG=Off-pump coronary artery bypass graft; PTCA=percutaneous transluminal coronary angioplasty; PVD=peripheral vascular disease; SD=standard deviation

**Table 2:** Demographic comparison between patients with and without preoperative beta blockers (n=282).

Variable	BB (197)	No BB (85)	*P*
Age (SD)	65.2 (9.6)	66.5 (9.3)	0.302
Female (%)	53 (26.9)	22 (25.9)	0.859
Smoker (%)	45 (22.8)	26 (36.6)	0.169
Hypertension (%)	154 (78.2)	68 (80)	0.731
Diabetes (%)	71 (36)	38 (44.7)	0.170
Dyslipidemia (%)	170 (86.3)	73 (85.9)	0.927
PVD (%)	8 (4.1)	4 (4.7)	0.806
COPD (%)	2 (1)	5 (5.9)	0.016*
Stroke (%)	7 (3.6)	4 (4.7)	0.646
AMI (%)	__	__	
Preoperative creatinine (mg/dl)	1.06 (0.42)	1.13 (1.14)	0.444
LVEF (%)	55.8 (11.4)	54.2 (13.9)	0.306
Previous PTCA (%)	28 (14.2)	14 (16.5)	0.625
Previous CABG (%)	3 (1.5)	__	0.253
NYHA III-IV (%)	8 (7.2)	10 (21.2)	0.042
Angina CCS III	46 (25)	21 (25.9)	0.638
Vessel disease (%)			0.139
1	7 (3.6)	3 (3.5)	
2	25 (12.7)	18 (21.2)	
3	158 (80.2)	58 (68.2)	
>1 IMA bypass (%)	54 (27.4)	21 (24.8)	0.893
OPCABG (%)	9 (10.6)	48 (24.4)	0.008*

AMI=acute myocardial infarction; BB=beta-blockers; CABG=coronary artery bypass grafts; CCS=Canadian Cardiovascular Society; COPD=chronic obstructive pulmonary disease; IMA=internal mammary artery; LVEF=left ventricular ejection fraction; NYHA=New York Heart Association; OPCABG=Off-pump coronary artery bypass graft; PTCA=percutaneous transluminal coronary angioplasty; PVD=peripheral vascular disease; SD=standard deviation

### Primary Outcome

**Fig. 1:**
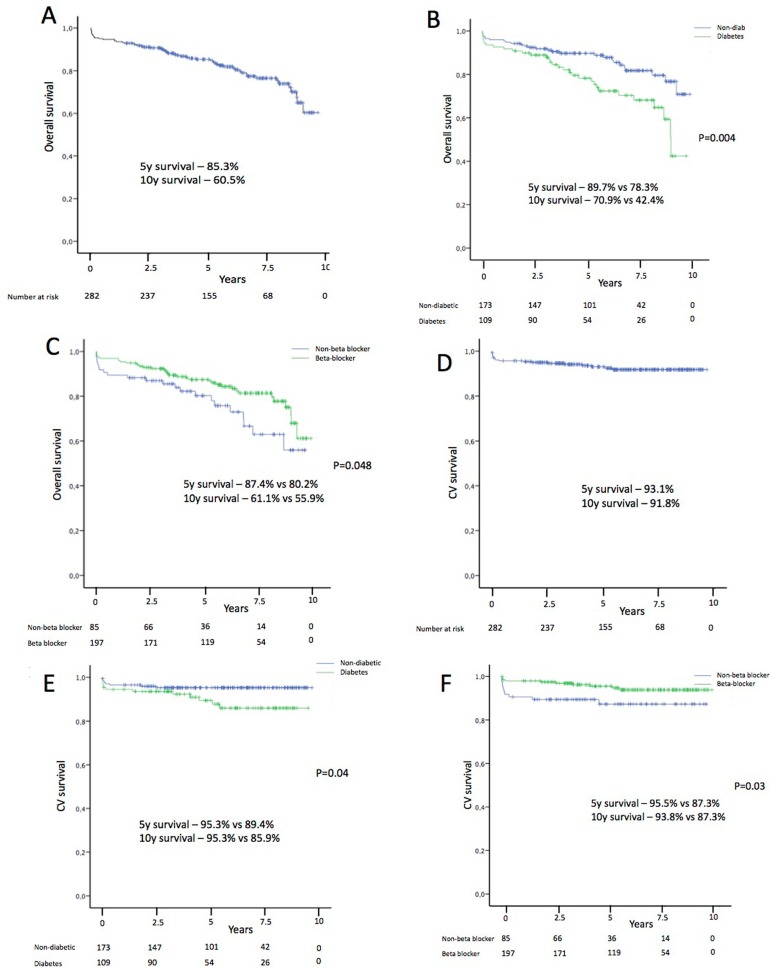
Survival of patients with stable angina after coronary artery bypass grafts (CABG). Overall survival (A, B, and C) and cardiovascular (CV) survival (D, E, and F). Global population (A and D), diabetic and non-diabetic patients (B and E), with beta-blocker and without beta-blocker treatment before surgery (C and F).

**Fig. 2:**
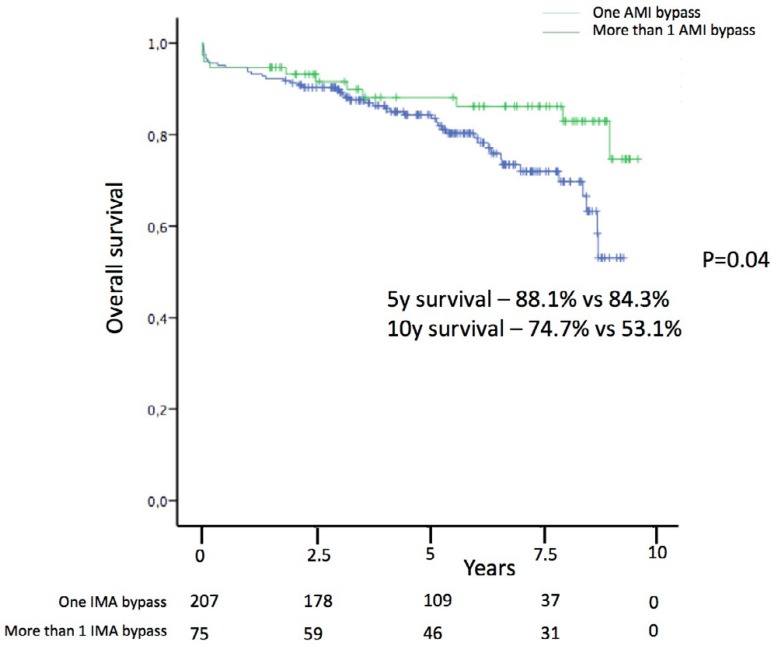
Overall survival in patients with stable angina after coronary artery bypass grafts (CABG) according to number of internal mammary artery (IMA) bypass done. AMI = acute myocardial infarction.

Overall 5 and 10-year survivals in the whole population were 85.3% (95% confidence interval [CI]: 81.0-89.6%) and 60.5% (95%CI: 50.3-70.7%), respectively. 5 and 10-year cardiovascular survivals in the whole population were 93.1% (95%CI: 90.096.2%) and 91.8% (95%CI: 88.3-95.3%), respectively. Mean overall survival (7.9 years, 95%CI: 7.3-8.7 years) in diabetic patients was significantly lower compared to non-diabetic patients (9.2 years, 95%CI: 8.7-9.7 years; *P*=0.004). Similar differences were obtained for cardiovascular survival in diabetic (9.3 years, 95%CI: 9.6-9.9 years) and non-diabetic patients (10.1 years, 95%CI: 9.8-10.4; *P*=0.038). Patients with preoperative use of beta-blocker therapy had better overall survival (9.0 years, 95%CI: 8.6-9.5) than those without treatment (7.9 years, 95%CI: 7.1-8.8 years; *P*=0.048). Cardiovascular survival was also improved with preoperative use of beta-blockers (10.1 years, 95%CI: 9.8-10.4 *vs.* 9.1 years, 95%CI: 8.4-9.8 years; *P*=0.03) (Figure 1). Patients with hypertension (8.5 years, 95%CI: 8.0-8.9 *vs.* 9.5 years, 95%CI: 8.9-10.1; *P*=0.011) were found to have worse long-term overall survival. More than one bypass (multiple) using IMA was associated with improved long term overall survival (9.3 years, 95%CI: 8.6-10.0 years *vs.* 8.3 years, 95%CI: 7.9-8.8 years; *P*=0.04) (Figure 2).

Predictors for overall survival after Cox regression analysis were: hypertension (hazard ratio [HR] = 2.60, 95%CI: 6.71-1.01), diabetes (HR = 2.07, 95%CI: 3.57-1.20), and age (HR = 1.06, 95%CI:1.03-1.09) (Table 3). Predictors for cardiovascular survival after Cox regression were: use of beta-blockers (HR = 0.43, 95%CI: 1.00-0.18), diabetes (HR = 2.36, 95%CI: 5.93-1.04), and age (HR = 1.08, 95%CI: 1.03-1.13) (Table 4).

**Table 3:** Predictors for overall survival in patients who underwent CABG for stable angina (n = 282).

Predictors	HR (95% CI)	*P*
Hypertension	2.60 (6.71-1.01)	0.049
Diabetes	2.07 (3.57-1.20)	0.009
Age	1.06 (1.03-1.09)	<0.001

CABG=coronary artery bypass grafts; CI=confidence interval; HR=hazard ratio

**Table 4:** Predictors for cardiovascular survival in patients who underwent CABG for stable angina (n=282).

Predictors	HR (95% CI)	*P*
Use of beta-blockers	0.43 (1.00-0.18)	0.05
Diabetes	2.36 (5.88-1.04)	0.05
Age	1.08 (1.03-1.13)	<0.001

CABG=coronary artery bypass grafts; CI=confidence interval; HR=hazard ratio

### Secondary Outcomes

Operative mortality during the study period was 3.5%. The risk for postoperative complications including stroke was very low (Table 5).

**Table 5:** Postoperative outcomes of patients who underwent CABG for stable angina (n=282).

Postoperative outcome	Patients (282)
Operative mortality (%)	10 (3.5)
Hemodialysis (%)	2 (0.7)
Pneumonia (%)	4 (1.4)
Stroke (%)	4 (1.4)
TIA (%)	17 (6)

CABG=coronary artery bypass grafts; TIA=transient ischemic attack

As we mentioned previously, diabetic patients had worse overall and cardiovascular survivals. When we analyzed only these patients, we found that use of beta-blockers (HR = 0.39, 95%CI: 0.81-0.19), female gender (HR = 2.71, 95%CI: 1.28-5.74), and age (HR = 1.07, 95%CI: 1.02-1.13) were predictors for overall survival (Table 6). Due to the low number of patients, we did not perform Cox regression for cardiovascular survival.

**Table 6:** Predictors for overall survival in diabetic patients who underwent CABG for stable angina (n=109).

Predictors	HR (95% CI)	*P*
Use of beta-blockers	0.39 (0.81-0.19)	0.012
Female	2.71 (1.28-5.74)	0.009
Age	1.07 (1.02-1.13)	<0.004

CABG=coronary artery bypass grafts; CI=confidence interval; HR=hazard ratio

## DISCUSSION

Revascularization in high-risk patients with acute coronary syndromes (ACS) (with or without ST-segment elevation) provides the best outcome with a significant reduction in death and myocardial infarction^[9]^. Conversely, the benefit of revascularization among patients with chronic stable CAD has been called into question^[10]^. Our study included patients with stable angina in whom more than 75% had three-vessel disease scheduled for isolated CABG. We were able to show that longterm overall and cardiovascular survivals after CABG in these patients were excellent. Operative mortality and postoperative complications were very low. Age, diabetes, and hypertension were found to be predictors for overall survival. Age, diabetes, and use of beta-blockers previous to CABG were found to be predictors for cardiovascular survival. In diabetic patients, the preoperative use of beta-blockers had a strong protective effect.

CAD remains the leading cause of mortality in most industrialized countries, although age-standardized mortality related to this condition has decreased by more than 40% during the last two decades^[11]^. Half of this decline resulted from prevention and reduction in major risk factors, whereas the other half has been attributed to medical treatment and revascularization^[12]^.

Previous data has shown that the percentage of patients who are free from angina after revascularization is much higher than those who remain with medical treatment exclusively^[13]^. Extensive review of the literature reveals that in developed countries, adherence to therapies averages 50%^[14]^. Therefore, revascularization represents an important adjunct to the global treatment of a patient with stable angina.

Long-term overall survival in our included patients was found to be similar to the data presented by the ASCERT trial^[15]^. Most of our patients died from non-cardiovascular causes, since the 10-year survival was 91.8%.

Predictors for overall survival found in our study were similar as those already published^[16]^. Hypertension and diabetes increased 2.6 and 2 times, respectively, the risk for overall mortality. Survival curves in diabetic and non-diabetic patients after surgery are parallel until 3 years after surgery when both diverge. This may be due to the diminished long-term permeability of vein grafts in diabetic patients^[17]^. Even though diabetes increases 2.3 times the risk for cardiovascular survival, the 10-year cardiovascular survival is excellent (85.9%). In contrast to our findings, other authors have shown than diabetes *per se* is not an independent predictor for poor prognosis^[18]^.

The benefit of preoperative use of beta-blockers in patients undergoing CABG has been recently debated. Ferguson et al.^[19]^ evaluated results from the Society of Thoracic Surgeons National Adult Cardiac Surgery Database, including patients who underwent surgery between 1996-1998, and they found that the preoperative use of beta-blockers is associated with an improvement in survival. Some years later, Brinkman et al.^[20]^ used the same database, including patients operated between 20082012, and found no survival benefit of preoperative use of betablockers. Both studies differ in two main issues: preoperative use of beta-blocker (50-60% in the study by Ferguson et al.^[19]^ and 86% in the study by Brinkman et al.^[20]^) and operative mortality (3% in Ferguson et al.^[19]^ and 1% in Brinkman et al.^[20]^). Probably, the significantly higher preoperative use of beta-blockers and lower mortality in the study by Brinkman et al.^[20]^ render it un-powered to show a survival benefit with beta-blockers. Rossi Neto et al.^[21]^ have shown that the preoperative use of metoprolol is associated with lower levels of troponin I, but no data regarding survival is presented. Our study reflects the outcome of a single center where almost 70% of patients were using beta-blockers previous to CABG. The preoperative use of beta-blockers was found to be associated with improved overall survival in the unadjusted model and remained an important predictor for cardiovascular survival after adjusting for other covariates. Its use was associated with a two-fold increase in cardiovascular survival. Analyzing survival curves, the beneficial effect of the preoperative use of beta-blockers occurs in the first months after surgery. Afterwards, both curves are almost parallel. Diabetic patients were found to be the main subgroup of patients who obtained benefit from preoperative use of beta-blockers. Although the beneficial use of beta-blockers on survival of patients with chronic CAD have already been shown^[22]^, the favorable outcome associated with preoperative use in patients scheduled for CABG is novel and has not been previously shown.

Use of multiple arterial bypass has shown to improve survival in patients after CABG^[23]^. This beneficial effect has been shown specially in diabetic patients^[24]^. We have found that patients who received more than one arterial bypass with IMA had improved overall survival. Looking at survival curves, the beneficial effect of multiple IMA bypass starts approximately five years after surgery. This may be due to the differential permeability in venous and arterial conduits after five years. Unluckily, follow-up angiography was not performed in our patients and therefore firm conclusions regarding this issue cannot be drawn.

### Limitations

Our study is subjected to limitations of retrospective studies, such as treatment bias and patient selection.

Although 282 patients might seem to be a low number of procedures, we were very strict in the database selection, restricting the inclusion only to patients with stable angina (no unstable nor acute myocardial infarction [AMI] scenarios) and isolated CABG. More than 70% of our CABG cases are acute coronary syndromes.

Due to the lack of governmental long-term follow-up of patients who underwent CABG, information regarding longterm adherence to prescription is limited. Nonetheless, we had 100% of adherence in the first month after surgery. Since survival curves in patients who took beta-blockers previous to CABG diverged mainly in the first few months after surgery and remained parallel afterwards, differences in long-term adherence to prescriptions would probably not explain these results.

The number of patients who underwent multiple IMA bypass was too low to do further analysis regarding its beneficial effect in diabetic population.

As a retrospective study, the strict use of beta-blockers preoperatively is uncertain and therefore this might have influenced the survival results.

## CONCLUSION

CABG is associated with excellent long-term overall and cardiovascular survivals in patients with stable angina. Among the already known predictors of survival, the preoperative use of beta-blockers was found to be protective mainly in patients with diabetes. These results should reinforce the role of CABG in the global treatment of patients with stable angina.

**Table:** 

Authors' roles & responsibilities	
VD	Substantial contributions to the conception or design of the work; or the acquisition, analysis, or interpretation of data for the work; drafting the work or revising it critically for important intellectual content; agreement to be accountable for all aspects of the work in ensuring that questions related to the accuracy or integrity of any part of the work are appropriately investigated and resolved; final approval of the version to be published
DP	Substantial contributions to the conception or design of the work; or the acquisition, analysis, or interpretation of data for the work; final approval of the version to be published
ES	Substantial contributions to the conception or design of the work; or the acquisition, analysis, or interpretation of data for the work; final approval of the version to be published
GS	Substantial contributions to the conception or design of the work; or the acquisition, analysis, or interpretation of data for the work; final approval of the version to be published
JE	Drafting the work or revising it critically for important intellectual content; final approval of the version to be published
